# Bio-catalytic degradation of dibenzothiophene (DBT) in petroleum distillate (diesel) by *Pseudomonas* spp.

**DOI:** 10.1038/s41598-023-31951-8

**Published:** 2023-04-13

**Authors:** Olawumi Oluwafolakemi Sadare, Michael Olawale Daramola

**Affiliations:** Department of Chemical Engineering, Faculty of Engineering, Built Environment and Information Technology, University of Pretoria, Hatfield, Pretoria, 0028 South Africa

**Keywords:** Biotechnology, Engineering

## Abstract

Biodesulfurization (BDS) was employed in this study to degrade dibenzothiophene (DBT) which accounts for 70% of the sulfur compounds in diesel using a synthetic and typical South African diesel in the aqueous and biphasic medium. Two *Pseudomonas* sp. bacteria namely *Pseudomonas aeruginosa and Pseudomonas putida* were used as biocatalysts. The desulfurization pathways of DBT by the two bacteria were determined by gas chromatography (GC)/mass spectrometry (MS) and High-Performance Liquid Chromatography (HPLC). Both organisms were found to produce 2-hydroxy biphenyl, the desulfurized product of DBT. Results showed BDS performance of 67.53% and 50.02%, by *Pseudomonas aeruginosa* and *Pseudomonas putida*, respectively for 500 ppm initial DBT concentration. In order to study the desulfurization of diesel oils obtained from an oil refinery, resting cells studies by *Pseudomonas aeruginosa* were carried out which showed a decrease of about 30% and 70.54% DBT removal for 5200 ppm in hydrodesulfurization (HDS) feed diesel and 120 ppm in HDS outlet diesel, respectively. *Pseudomonas aeruginosa* and *Pseudomonas putida* selectively degraded DBT to form 2-HBP. Application of these bacteria for the desulfurization of diesel showed promising potential for decreasing the sulfur content of South African diesel oil.

## Introduction

Diesel has been regarded as the larger and most widely used source of energy in the world. It consists of sulfur compounds that when combusted by direct method release toxic substances such as sulfur oxides (SO_x_) into the atmosphere which contribute to acid rain, health diseases, and environmental pollution^1^. The world health organization recorded about 7 million deaths in 2012 attributed to air pollution^2^, associated with particulate matter. For instance, the South African government requires that the concentration of sulfur compounds in diesel should be ≤ 10 ppm^3^. Beginning in January 2006, South Africa has two types of diesel fuel available. The maximum sulfur content for standard-grade diesel is 500 ppm, compared to 50 ppm for low sulfur grade fuel. Nevertheless, Sasol has been providing 10 ppm diesel to automobiles used for business and personal purposes^4^. In addition, the South African government has issued rules guiding the minimum allowable sulfur content in the country’s diesel fuel, which may propel the upgrading of diesel products by some oil refineries so as to meet the criteria^5^. This regulation will take effect from September 2023. An effective desulfurization approach can consecutively enhance the quality of the fuel, with respect to the cetane value and increase in octane number^6^. Therefore, the interest of researchers has been provoked in this direction to further reduce the sulfur contents in South African diesel.

Different techniques such as hydrodesulfurization, adsorption^7^, oxidation, extraction, and biodesulfurization have been studied by researchers to reduce the amount of sulfur-containing compounds in petroleum distillates^8^. Conventional hydrodesulfurization (HDS) is the most commonly used technique in the refineries to reduce the sulfur content of diesel with huge success recorded. Though HDS is capable of reducing sulfur content in diesel such as heterocyclic sulfur compounds like thiols and sulfides, the technique is inefficient to desulfurize refractory sulfur compounds such as dibenzothiophene and its derivatives, especially 4,6-dimethyldibenzothiophene (4,6-DMDBT)^9^. In addition, this technology suffers from high capital and operating costs^10^ due to the fact that it is operated at high temperature and pressure, thereby making it energy-intensive^11^. Furthermore, a lot of hydrogen is used, making the process highly risky in terms of safety. This situation has provoked the thoughts of many researchers worldwide to urgently explore alternative routes for the desulfurization of petroleum distillates.

Biodesulfurization (BDS) is a promising method that could be an efficient, cheap, and less energy-intensive technique for desulfurization^7^. However, BDS has not met the industrial-scale application’s requirements yet^12^. For BDS to attain full industrial acceptance for utilization in petroleum desulfurization, the desulfurization bacteria must possess major characteristics such as; high tolerance for solvents, high desulfurization efficiency, and wide substrate specificity^13^. BDS involves the use of biocatalysts for the degradation of the sulfur compound in petroleum distillate to a less harmful compound without altering the quality of the fuel. Different bacteria strains such as *Rhodococcus* sp.^14,15^, *Pseudomonas* sp.^16^, and *Gordona* sp.^17^ have been employed for degrading sulfur compounds in diesel oil. For instance, *Pseudomonas strains* used in this study are very prolific and could survive in biphasic media and metabolic diversity^18^. They are known to thrive in conditions with or without oxygen, although they are classified as aerobic. They are readily available since they are present naturally in soil and water.

In BDS, only C-S oxidative bond cleavage takes place to release the sulfur atom as sulfate and the carbon skeleton of the thiophenic compound remains unaffected as a phenolic product. Consequently, in the BDS process, the thiophenic sulfur compound serves only as the sole sulfur source for bacteria growth, and the calorific value of the fuel is preserved by the final end product 2-HBP^19–21^. A variety of sulfur-containing compounds have been reported, but dibenzothiophene (DBT) (the recalcitrant sulfur compound during HDS) accounts for 70% of the sulfur compounds in diesel and it is considered a model compound for biodesulfurization in research^22,23^. Various investigations have been reported on the use of these *pseudomonas strains* differently, under different conditions. Al-Faraas et al.^24^ isolated *Pseudomonas aeruginosa* from Iraqi Soils for desulfurization of dibenzothiophene. The effect of different sulfur sources on the degradation efficiency of *Pseudomonas spp.* has been reported. However, reports on detailed desulfurization activity of the bacteria are limited in the literature^24^. Davoodi-Dehaghani et al.^25^ also investigated the desulfurization ability of *Rhodococcus Erythropolis* (SHT87) for the degradation of DBT. The results showed that about 3 mM DBT was totally metabolized by the SHT87 resting cells in the biphasic and aqueous systems within 10 h. The strain was able to make use of thiophene, dimethylsulfoxide, dibenzothiophene sulfone, 2- methylthiophene and DBT, as the only sulfur sources for growth at 30 °C^25^. Mingfang et al.^26^ desulfurize DBT and 4, 6-dimethyldibenzo thiophene in dodecane and straight run diesel using lyophilized cell of *Pseudomonas delafieldii* R-8. Results showed about 1,807 mg/L of sulfur desulfurized in the straight-run diesel oil. The specific desulfurization rate was found to be 8.75 mmol sulfur kg^−1^ (cell) h^−1^. Furthermore, Bhanjadeo et al.^27^ explored the desulfurized potential of Microbial Type Culture Collection (MTCC) strains on DBT via C-S bond cleavage (4-S pathway). The study revealed > 99% DBT desulfurization within 10 min^27^. As far as it could be ascertained, no recent studies have been reported on the biodegradation of dibenzothiophene in South African diesel using *Pseudomonas strains*.

BDS is considered a complementary technique to the HDS method of desulfurization. It can also be used to develop a hybrid process (Adsorption/BDS) to remove sulfur-containing compounds from petroleum distillates. In order to achieve this goal, there is a need to investigate the effect of operating conditions for biodesulfurization using the *Pseudomonas* sp. Therefore, this study investigates and compares the biodegradation efficiencies of growing and resting cells of *Pseudomonas aeruginosa* and *Pseudomonas putida* on the sulfur compound (DBT) in a model diesel and typical South African diesel in the aqueous and biphasic medium.

## Materials and methods

*Isolated Pseudomonas putida and Pseudomonas aeruginosa* Kwik Stik, ATCC 27,853 Microbiologics, were purchased from Sigma Aldrich (Pty) Ltd., South Africa. Hydrodesulfurizer inlet feed (5200 ppm) and hydro-treated diesel (120 ppm) were obtained from a South African refinery. Dibenzothiophene, acetonitrile and dimethyl formamide (purity, GC) (99%) were purchased from Merck (Pty) Ltd., South Africa. Ethyl acetate was purchased from NT laboratories supply (Pty) Ltd., South Africa. Glycerol, hexadecane, and 2-hydroxylbiphenyl (2-HBP) were purchased from Sigma-Aldrich (Pty) Ltd, South Africa. All other chemicals were of analytical grade, commercially available and used without further purification. All methods were performed in accordance with the relevant guidelines and regulations.

### Preparation of basal salt medium (BSM)

Basal salt medium consisting of NaH_2_PO_4_·H_2_O (4 g/L), K_2_HPO_4_·3H_2_O (3 g/L), MgCl_2_·6H_2_O (0.0245 g/L), CaCl·2H_2_O (0.001 g/L), FeCl_3_·6H_2_O (0.001 g/L) was prepared in the laboratory as described by Boltes et al.^16^. All glass wares were sterilized in an autoclave at 121 °C, for 20 min. The chemicals were dissolved in deionized water until all chemicals were dissolved. The pH of the medium was maintained at 7.0 by adding 0.5 M NaOH, drop-wisely. The solution was sterilized in autoclave at the same conditions as for the glassware mentioned earlier. The prepared BSM medium was stored at room temperature and kept away from sunlight.

### Preparation of inoculums

Frozen pellets of *Pseudomonas aeruginosa* and *Pseudomonas putida* each were inoculated in 50 mL Luria–Bertani (LB) liquid medium in a 250 mL Erlenmeyer flask. 10 g/L of tetracycline was prepared and 150 µL was added to the inoculum to serve as antibiotics for the bacteria. The mixture was incubated for 10 days at 30 °C, and 37 °C for *Pseudomonas putida* and P*seudomonas aeruginosa*, respectively and agitated at 130 rpm. The prepared inoculum was kept in an eppendorf tube and frozen at − 80 °C.

### Biodesulfurization (BDS) experiment with growing cells of *Pseudomonas aeruginosa* and *Pseudomonas putida*

The BDS experiments were conducted in batch mode. The bacteria of *Pseudomonas putida and Pseudomonas aeruginosa* previously inoculated in LB medium was used. The experiment was conducted as described by Al-Faraas et al.^24^ and Boltes et al.^15^. 0.25 mL LB-frozen stock of bacteria was put into 50 mL of BSM in a 250 mL Erlenmeyer flask. 0.1 g of DBT was dissolved in dimethyl formamide (DMF) and serially diluted to vary the concentration as required. The bacteria medium was supplemented with 1 mL of 0.25 mM (46 ppm) DBT as the only sulfur source with 150 µL tetracycline. Glycerol 20 g/L was used as the only carbon source. The flask was incubated at 30 °C and 37 °C for *Pseudomonas putida* and *Pseudomonas aeruginosa*, respectively, and agitated at 130 rpm for 10 days. The growth of bacteria was measured and desulfurization of DBT in model diesel was monitored as well.

### Biodesulfurization experiment with resting cells of *Pseudomonas aeruginosa* and *Pseudomonas putida*

Biodesulfurization with resting cells of *Pseudomonas aeruginosa* and *Pseudomonas putida* was performed in batch mode. The bacteria in the growing cell experiment were harvested at the late exponential phase. The bacteria medium was centrifuged at 7000 rpm for 5 min. The cells were washed thrice with potassium phosphate buffer solution. The washed *Pseudomonas aeruginosa* and *Pseudomonas putida* cells were then re-suspended into glycerol/ NaCl in the ratio of 1:1. The different cell concentrations (0.3–1.2 g DCW/L) of frozen resting cells of *Pseudomonas aeruginosa* and *Pseudomonas putida* in glycerol/NaCl solution were measured into 50 mL of BSM with 150 µL of tetracycline, 500 µL of glycerol and 1 mL of 500 ppm model oil. The initial concentrations of DBT varied from 250 to 1000 ppm. The mixture was incubated for 8 h at 30 °C and 37 °C for *Pseudomonas putida and Pseudomonas aeruginosa,* respectively, and the mixture was agitated at 130 rpm. Aliquots of samples were taken at intervals and the samples were analyzed for biodesulfurization efficiency.

Resting cells of *Pseudomonas aeruginosa* and *Pseudomonas putida* were used for the degradation of real diesel in this study. About 5 mL of diesel was measured into a 250 mL Erlenmeyer flask with 5 mL of resting cell in glycerol/NaCl (1:1) into 20 mL of BSM. The solution was incubated for 8 h, 130 rpm at 30 °C for Pseudomonas putida and 37 °C for Pseudomonas aeruginosa, and aliquots of samples were taken at intervals for analysis.

### Aqueous and biphasic media experiment

Hexadecane was chosen as an organic phase in the biphasic process owing to its presence in the diesel oil fraction. BSM was the aqueous medium. In this experiment, the percentage of oil-to-water varied from 0%, 20%, and 50%. The organic phase was centrifuged and extracted with ethyl acetate from the aqueous phase oil. The final DBT concentration and produced 2-HBP end product of the 4S pathway in the organic phase were determined according to Caro et al.^28^. This is explicitly described under the analytical techniques in Section “Analytical techniques for measurements of bacteria growth and desulfurization of model oil and typical real diesel during biodesulfurization experiments”.

### Desulfurization efficiency of *Pseudomonas* strains on real South African diesel

Resting cells of *Pseudomonas aeruginosa* and *Pseudomonas putida* were used for degradation of real diesel in this study. Diesel sample obtained before HDS with initial DBT concentration of 5200 ppm and diesel sample obtained after HDS with initial DBT concentration of 120 ppm were supplied by a refinery in South Africa. About 5 mL of diesel was measures into a 250 mL Erlenmeyer flask with 5 mL of resting cell in glycerol/NaCl (1:1) and 20 mL of BSM. The mixture was incubated for 8 h, at 30 °C and 37 °C, and agitated at 130 rpm for *Pseudomonas putida* for *Pseudomonas aeruginosa, respectively.* Aliquots of samples were taken at intervals for analysis^29^.

### Analytical techniques for measurements of bacteria growth and desulfurization of model oil and typical real diesel during biodesulfurization experiments

The pH was measured using a pH meter and a Spectroquant Pharo 300 Merck, (W210324), made in the EU was used to measure the turbidity of the culture. The cell mass was determined by measuring the optical density (OD) at a wavelength of 660 nm. In order to measure the net dry cell weight (g DCW) of the biomass, 3 mL of the culture was centrifuged at 7000 rpm for 10 min. The concentrate was washed thoroughly on a pre-weighed filter paper. The filter paper containing the bacteria was dried overnight at 100 °C and the weight of the bacteria was determined by subtracting the net weight from the initial weight of the filter paper. The relationship between the optical density (OD_660 nm_), dry mass, and absorbance was determined. A calibration curve was obtained and used in the determination of the unknown cell concentration.

To obtain the final sulfur concentration in the desulfurized model oil, aliquots of samples were taken at intervals and the concentration of DBT was analyzed using Agilent High-Performance Liquid Chromatography (HPLC), equipped with an Eclipse C-18 column. Acetonitrile (55 wt%) was used as the mobile phase, with the UV detector at 254 nm, with a 1.0 mL/min flow rate, and injection volume of 10 mL for 10 min. The incubated mixture was centrifuged at 7000 rpm for 10 min and filtered. The aqueous phase was acidified with HCl in order to quench the desulfurization reaction before analyzing it with HPLC. The degraded DBT and the formed 2-HBP formed were extracted with an equal volume of ethyl acetate. In a biphasic system, the DBT and 2-HBP were extracted from the organic phase after centrifugation and analyzed using HPLC. The peak area of DBT and that of the 2-HBP of known concentrations at different elution time were used to calibrate the HPLC. The calibration curve obtained during the calibration was used to determine the unknown concentration of DBT and 2-HBP in the desulfurized samples.

Detection of sulfur containing compounds in diesel before hydrodesulfurization (5200 ppm) and diesel after hydrodesulfurization (120 ppm) and the evolution of the 4S end product (2-HBP) were done by gas chromatography/mass spectroscopy (GC/MS) Shimadzu equipment with column Rx-SMX. Injection and detection temperature were set at 220 °C and 230 °C, respectively. Oven temperature at 80 °C, to 190 °C at 10 °C/min and 15 °C/min to 230 °C for 18 min in split-less mode. Helium was used as the carrier gas. To determine the calibration curve for HDS feed, and HDS outlet diesel, the diesel samples with known concentrations were diluted serially to vary their concentrations. The peak areas detected from GC/MS were plotted against known concentrations of the DBT and a calibration curve was obtained which was used to calculate the unknown concentrations of DBT in the samples. Physical and chemical properties of a typical South African diesel oil is given in Table 1.

**Table 1:** Physical and chemical properties of a typical diesel fuel obtained from refinery X, South African.

Properties	Values
Total sulfur (ppm)	5200 HDS feed, 120 ppm HDS outlet
Density at 15 °C, kg/m^3^	820.0
Ignition point	> 55
Cetane index	46.0
Kinematic viscosity at 40 °C	3.98
Polycyclic aromatic hydrocarbon, wt%	2.1
V % distilled until 250 °C	< 40
Distillation volume % distilled until 300 °C	75
End of distillation, °C	342

## Results

### Growth of *Pseudomonas aeruginosa* and *Pseudomonas putida* with degradation of DBT and formation of 2-HBP as a function of time

**Figure 1:**
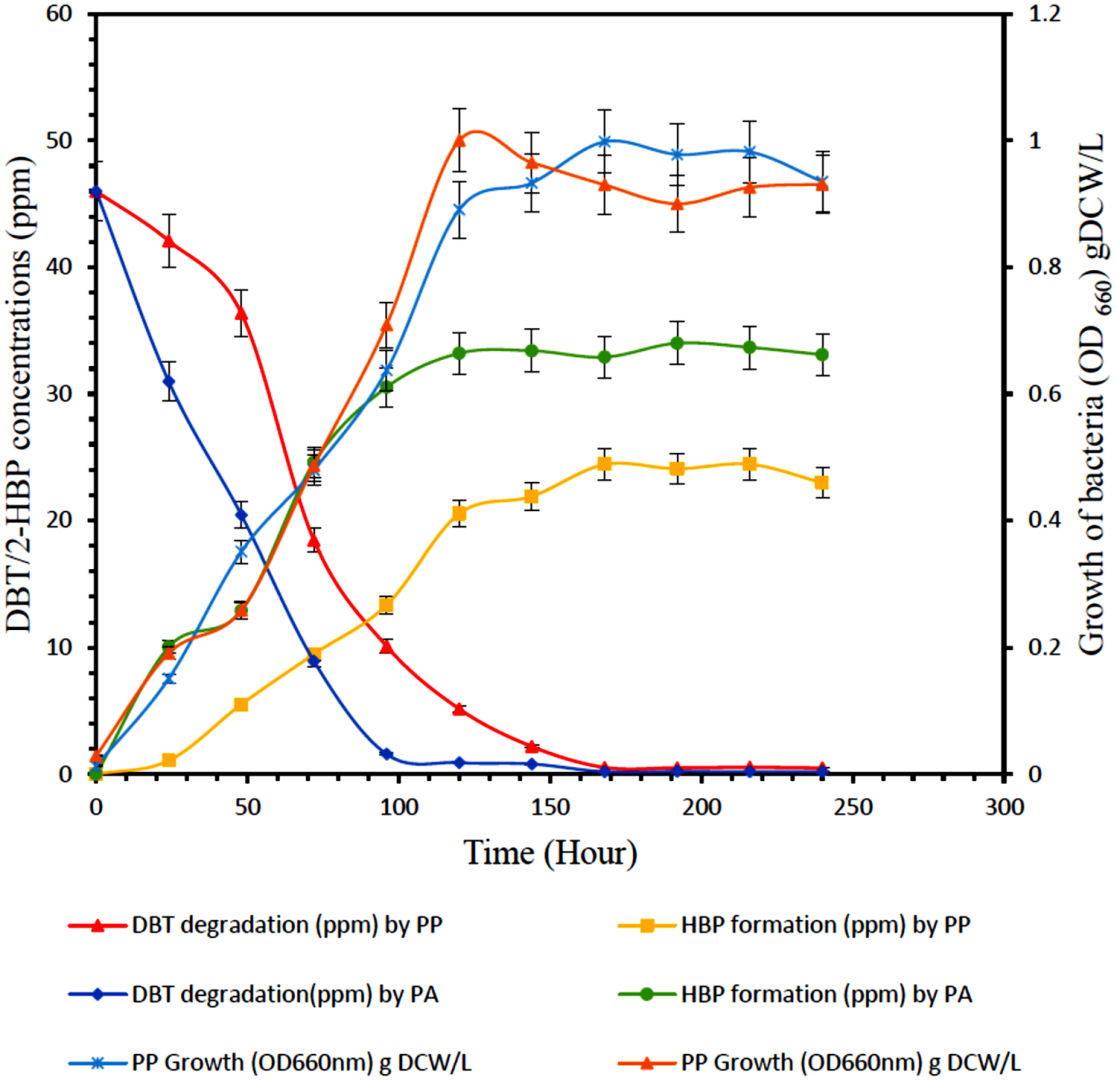
Cell growth of *Pseudomonas aeruginosa* and *Pseudomonas putida* with DBT degradation and 2-HBP formation. Experimental conditions: Initial DBT concentration 46 ppm, Temperatures 30 °C and 37 °C for *Pseudomonas aeruginosa and* P*seudomonas putida*, respectively, Shaking speed 130 rpm.

Figure 1 depicts the growth of P*seudomonas aeruginosa and Pseudomonas putida* with degradation of DBT and formation of 2-HBP as a function of time. The results showed that the growth of the bacteria increased with time. The growth of *Pseudomonas aeruginosa* began to decrease slightly after 120 h and the growth of P*seudomonas putida* began to decrease after168 h. At 120 h, an optical density of *Pseudomonas aeruginosa* reached 1.0 g DCW/L and that of *Pseudomonas putida* was at 0.998 g DCW/L. It can be observed that as the growth of bacteria increased there was a simultaneous increase in the degradation of DBT from 46 to 0.21 ppm for *Pseudomonas aeruginosa* and and from 46 to 0.51 ppm for *Pseudomonas putida*. Like wisely, the amount of 2-HBP increased from 0 to 32.5 ppm for PA, and the amount of 2-HBP increased from 0 to 24.5 ppm for PP. All experiments revealed that the growth of the bacteria ceased before the DBT was fully converted to 2-HBP. In addition, the production of 2-HBP, as a final metabolite of the 4S pathway, was less than the consumption of DBT in both cases. These results agree with the results reported by Davodii-Dehaghani et al.^25^ and Caro et al.^30^. This could be a result of intra and extracellular accumulation of 4S compounds. The desulfurization of DBT to 2-HBP through the 4S pathway could be the reason why there was no further growth in the bacteria *Pseudomonas aeruginosa* and *Pseudomonas putida*, at 120 h for *Pseudomonas aeruginosa* and 168 h for P*seudomonas putida*. This is due to the inhibition effect. In view of the report that the main limiting factor of BDS of dibenzothiophene is the inhibitory effect of 2-HBP.

The major 4S pathway metabolite identified during the batch cultivation was 2-HBP. This was also confirmed by Rhee et al.^22^. Approximately 99.5% and 98.9% of DBT was degraded by *Pseudomonas aeruginosa* and *Pseudomonas putida*, respectively. However, 2-HBP could accumulate up to concentration of 33.06 ppm for P*seudomonas aeruginosa* and 22.99 ppm for *Pseudomonas putida* which account for 71.9% and 50% formation of 2-HBP for *Pseudomonas aeruginosa* and *Pseudomonas putida*, respectively. The results show that the amount of 2-HBP formed was not equivalent to the amount of DBT degraded. In addition, no sulfate or sulfite accumulation was detected during growth of bacteria. Therefore, it could be assumed that the sulfur content has been assimilated by the cells.

**Figure 2:**
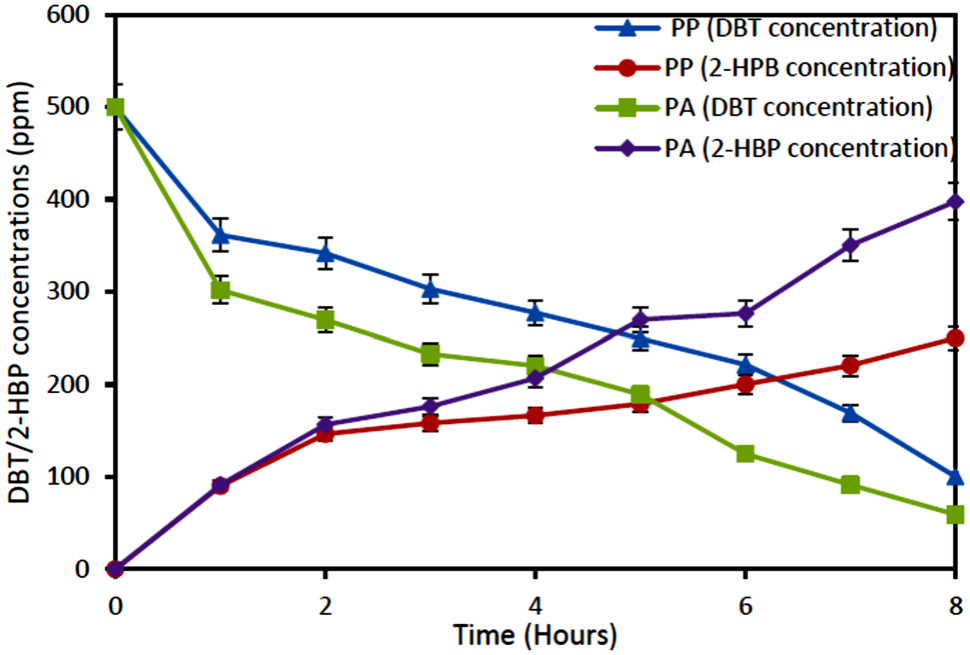
Degradation of DBT and formation of 2-HBP formation by resting cells of *Pseudomonas aeruginosa and pseudomonas putida.* Experimental conditions: Initial DBT concentration 500 ppm, Initial cell concentration 1.2 g DCW/L, Temperature 37 °C (Pseudomonas aeruginosa), 30 °C (Pseudomonas putida), Shaking speed 130 rpm.

Figure 2 depicts the degradation of DBT and formation of 2-HBP by *Pseudomonas aeruginosa* and P*seudomonas putida*. The result showed gradual degradation of DBT from 500 to 59.17 ppm for *Pseudomonas aeruginosa* and from 500 to 100.17 ppm for P*seudomonas putida*. Accumulation of 2-HBP also reached 250 ppm for P*seudomonas putida* and 397.67 ppm for *Pseudomonas aeruginosa*. This accounts for 88% and 80% desulfurizing capability of *Pseudomonas aeruginosa* and P*seudomonas putida*, respectively. The percentage of 2-HBP produced were 79.5% and 50% for *Pseudomonas aeruginosa* and *Pseudomonas putida*, respectively.

### Effect of operating variables on degradation of DBT and formation of 2-HBP in model diesel by *Pseudomonas aeruginosa* and *Pseudomonas putida*

**Figure 3:**
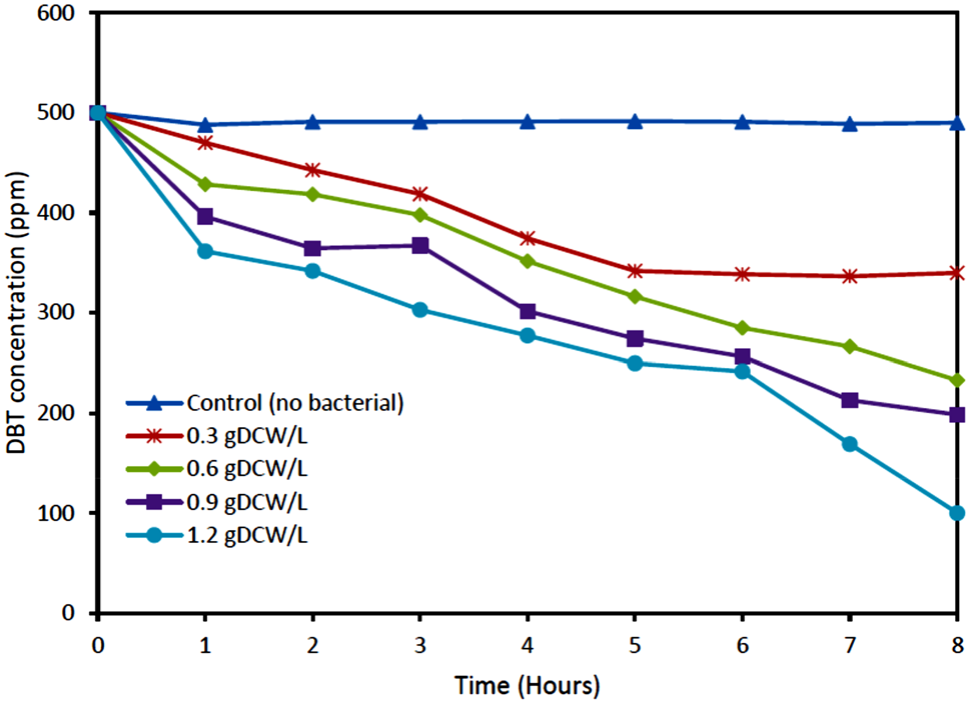
Effect of cell concentrations of *Pseudomonas putida* during degradation of DBT. Experimental conditions: Initial DBT concentrations 500 ppm, Temperature 30 °C, Shaking speed 130 rpm.

Figure 3 depicts the effect of cell concentration on biodesulfurization of DBT in the model oil by resting cells of *Pseudomonas putida* with respect to the control sample that has no bacteria*.* The concentration of cells varied from 0.3 to 1.2 g DCW/L and the initial DBT concentration was 500 ppm*.* The results showed the effect of bacteria concentration on the biodesulfurization of DBT. It could be observed that biodesulfurization of DBT increased with an increase in the concentration of cells from 0.3 to 1.2 gDCW/L compared to when there were no bacteria in the control sample. This is an indication that the bacteria strain used the DBT for their metabolism as the only sulfur source. No DBT degradation was noticed when there were no bacteria in the medium. The result showed the highest desulfurization capability of DBT when the cell concentration was 1.2 g DCW/L with 80% degradation effect on DBT. This could be as a result of more *Pseudomonas putida* in the medium to degrade the DBT compound*.*

**Figure 4:**
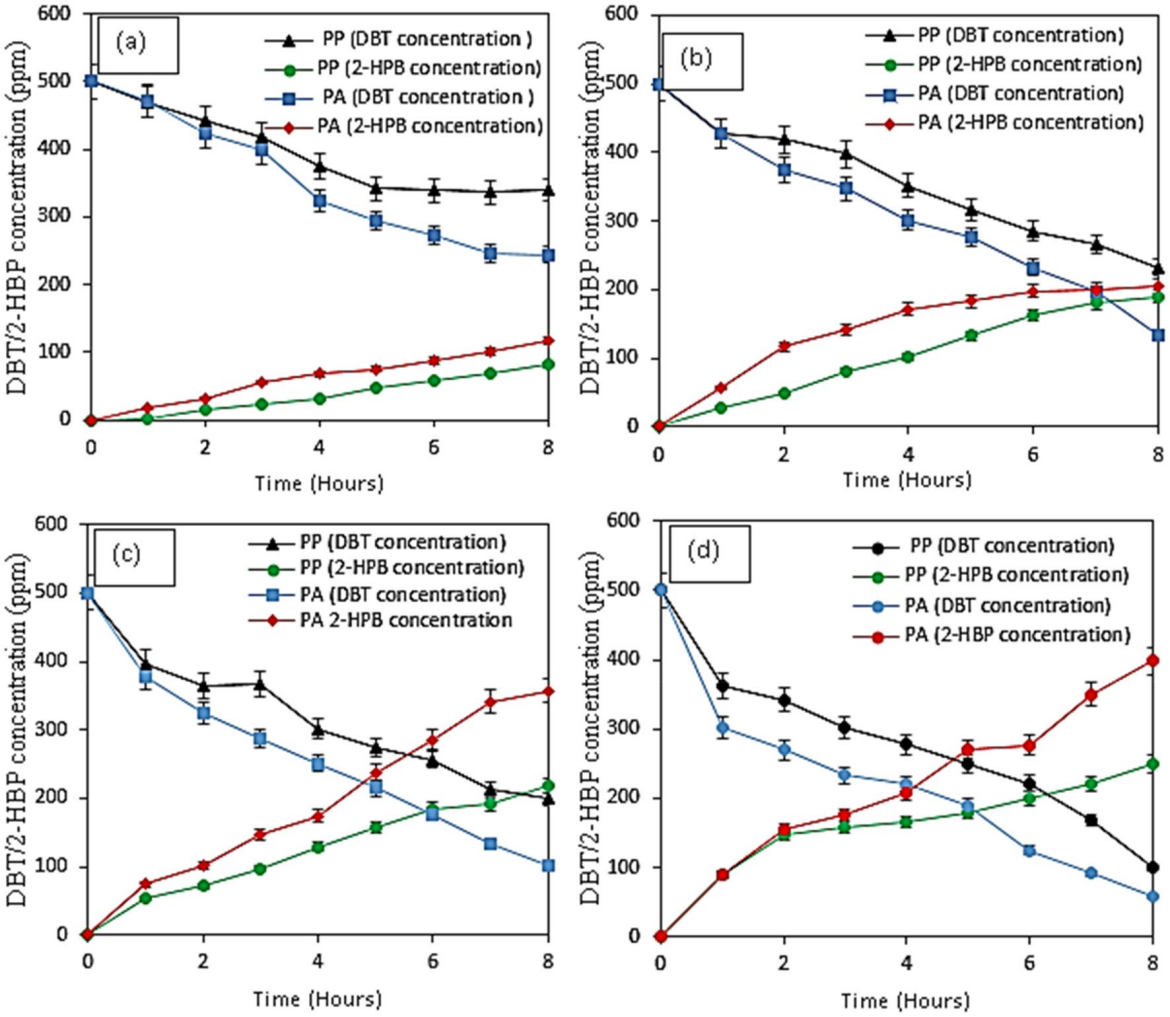
Effect of cell concentration of resting cells of *Pseudomonas putida* and *Pseudomonas aeruginosa* on the DBT degradation and production of 2-HBP (**a**) 0.3 g DCW/L (**b**) 0.6 DCW/L (**c**) 0.9 g DCW/L (**d**) 1.2 g DCW/L. Experimental conditions: Initial DBT concentrations 500 ppm, Temperature 37 °C (*Pseudomonas aeruginosa*) 30 °C (*Pseudomonas putida*), Shaking speed 130 rpm.

Desulfurization of DBT and the production of 2-HBP is illustrated by the results in Fig. 4. The results showed that as the cell concentration in the medium increased, desulfurization of DBT also increased, and production of 2-HBP is enhanced as presented in Table 2. This could be as a result of more available bacteria present to feed on the DBT, which in return increased the production of 2-HBP. The lowest desulfurizing capacity of 55% and 32% for *Pseudomonas aeruginosa* and *Pseudomonas putida*, respectively were achieved when cell concentration was 0.3 g DCW/L. In addition, 2-HBP produced for *Pseudomonas aeruginosa* and *Pseudomonas putida* at 0.3 g DCW/L was 117.34 ppm and 81.04 ppm, respectively, accounting for 23.47% and 16% *Pseudomonas aeruginosa* and *Pseudomonas putida*, respectively. The highest desulfurization performance was obtained when 1.2 g DCW/L was used. Enhancement in desulfurization of DBT and formation of 2-HBP at an increased concentration of bacteria could be attributed to the availability of more bacterial to feed on the DBT. It could be observed that formation of 2-HBP was lower than the degradation of DBT in all the cases. This could be attributed to the inhibition of bacteria growth as a result of the production of the 2-HBP^31^. It is a well-known fact that sulfate and 2-HBP, which are end products of DBT desulfurization, have direct adverse effects on the BDS. Therefore, enzymes of 4S pathway also undergo feedback inhibition exerted by 2-HBP, hence, limiting the cell growth, resulting in low desulfurization efficiency. These results obtained in this study agree with the findings reported by Mohebali and Ball^31^.

**Table 2:** Initial and final concentrations of DBT/2-HBP during biodesulfurization of DBT by *Pseudomonas aeruginosa* and *Pseudomonas putida.*

Cell conc. (g/DCW/L)	Initial conc. of DBT (ppm)	Final conc. of DBT (ppm)	Initial conc. of 2-HBP (ppm)	Final conc. of 2-HBP (ppm)
*Pseudomonas aeruginosa*				
0.3	500	244.00	0	117.34
0.6	500	132.80	0	205.34
0.9	500	100.40	0	357.57
1.2	500	59.17	0	397.67
*Pseudomonas putida*				
0.3	500	339.70	0	81.04
0.6	500	232.80	0	190.14
0.9	500	198.40	0	217.87
1.2	500	100.17	0	250.11

**Figure 5:**
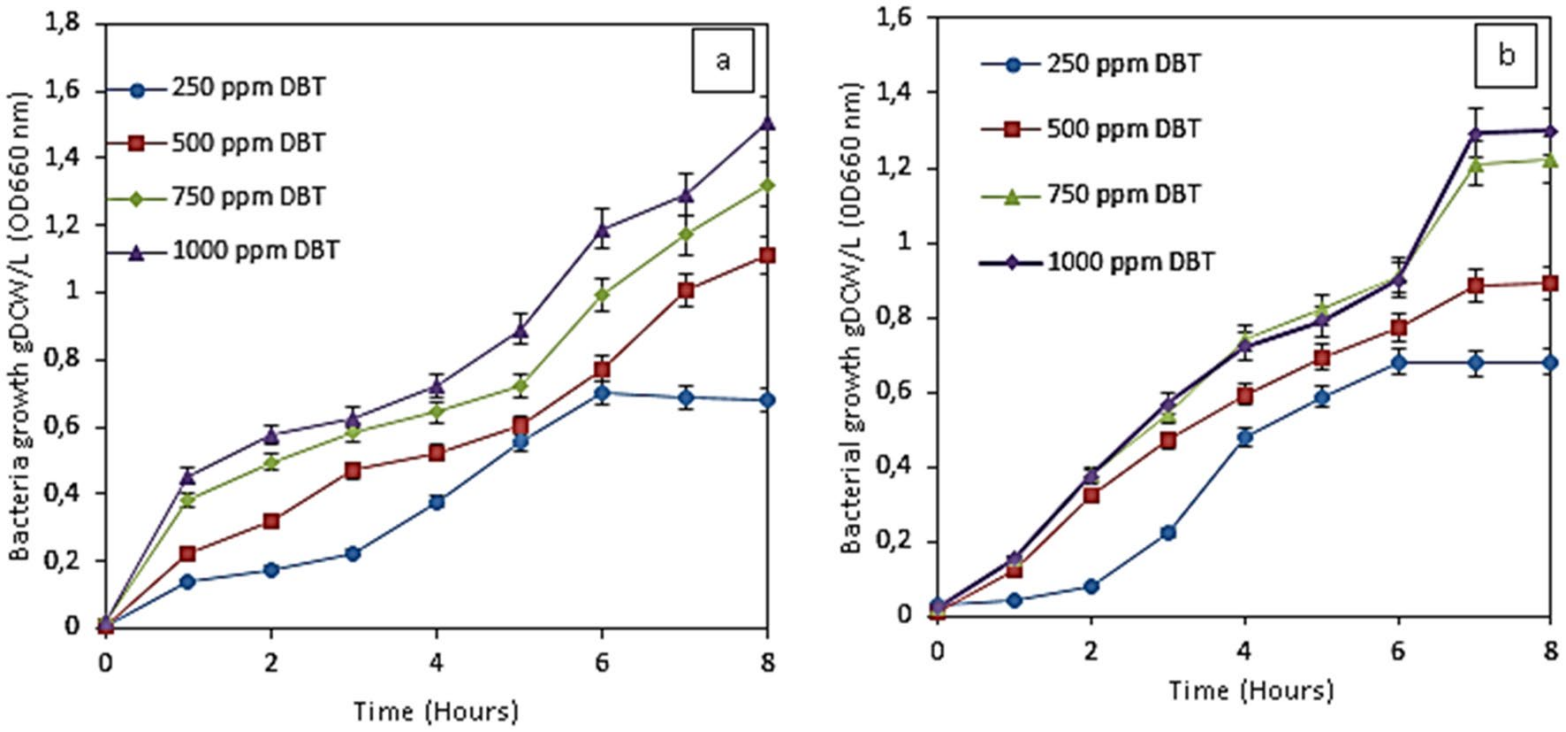
Effect of initial DBT concentrations on the growth of (**a**) *Pseudomonas aeruginosa* (**b**) *Pseudomonas putida* at 1:4 oil: water ratio for biphasic. Experimental conditions: Temperature 37 °C (*Pseudomonas aeruginosa*), 30 °C (*Pseudomonas putida*), Shaking speed 130 rpm.

Figure 5(a & b) depicts the effect of initial DBT concentration on the growth of *Pseudomonas aeruginosa* and P*seudomonas putida*. The initial concentrations of DBT varied from 250 to 1000 ppm. The results showed that an increase in the initial concentration of DBT resulted in an increase in the growth of bacteria (*Pseudomonas aeruginosa* and *Pseudomonas putida*). This could be a result of enough availability of DBT for the metabolism of the cell in the medium, resulting thereby in enhanced growth. It was discovered that growth stopped before the final or complete desulfurization of the DBT. This could be attributed to the accumulation of the 2-HBP compound in the medium that inhibited the further growth of the cells at 7 h as a result of the inhibition effect. This agrees with the result of Maxwell et al.^32^. Other DBT metabolites of the 4S pathway such as DBTO, DBTO2, and HPBS were not detected by GC–MS analysis, except 2-HBP. This could be attributed to the existence of an additional degradation pathway for DBT. Other authors also confirmed that other metabolites of the 4S pathway could not be detected in the experiment. However, they are indicated as postulated metabolites^32–34^.

**Figure 6:**
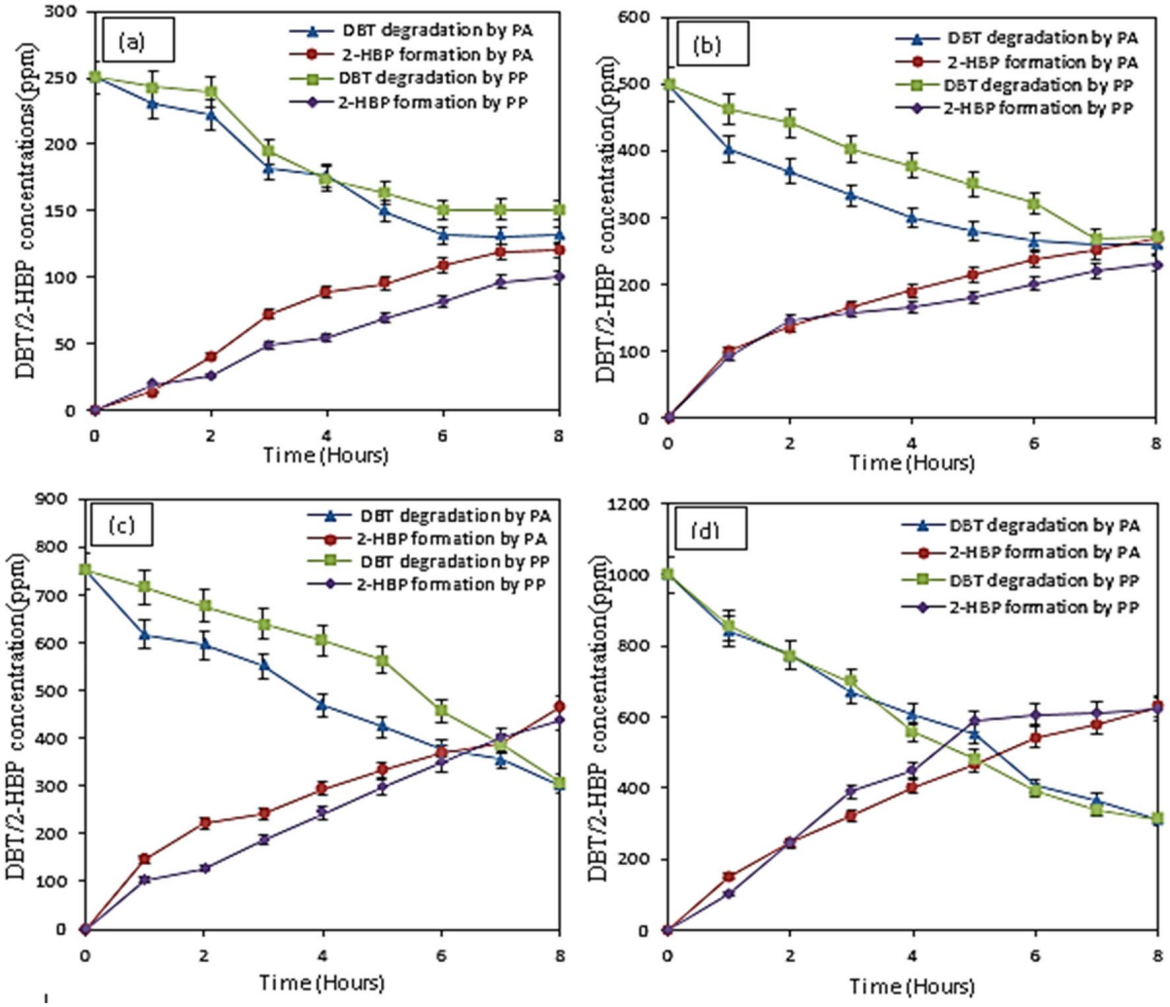
Effect of initial DBT concentration on DBT degradation by *Pseudomonas aeruginosa and Pseudomonas putida* (**a**) 250 ppm, (**b**) 500 ppm (**c**) 750 ppm (**d**) 1000 ppm. Experimental conditions: Cell concentration 1.2 g DCW/L, Temperature 37 °C (Pseudomonas aeruginosa), 30 °C (Pseudomonas putida), Shaking speed 130 rpm.

Figure 6(a & b) depicts the effect of DBT initial concentrations on the biodesulfurization of DBT by PA and PP. The initial concentration of DBT was varied from 250 to 1000 ppm, in order to evaluate their effect on the growth, DBT desulfurization, and 2-HBP formation. The results showed that an increase in the initial concentration of DBT increased the growth of bacteria, thereby increasing the biodesulfurization of DBT which also resulted in an increase in 2-HBP production. About 80%, 67.53%, 40%, and 32.97% BDS efficiencies were achieved at 250, 500, 750, and 1000 ppm, respectively, when *Pseudomonas aeruginosa* was used as the biocatalyst. About 60.06%, 50.02%, 38.71%, and 30.21% desulfurization efficiencies were achieved when *Pseudomonas putida* was used as the biocatalyst for 250 ppm, 500 ppm, 750 ppm, and 1000 ppm, respectively.

### Biphasic effect on growth and BDS desulfurization

**Figure 7:**
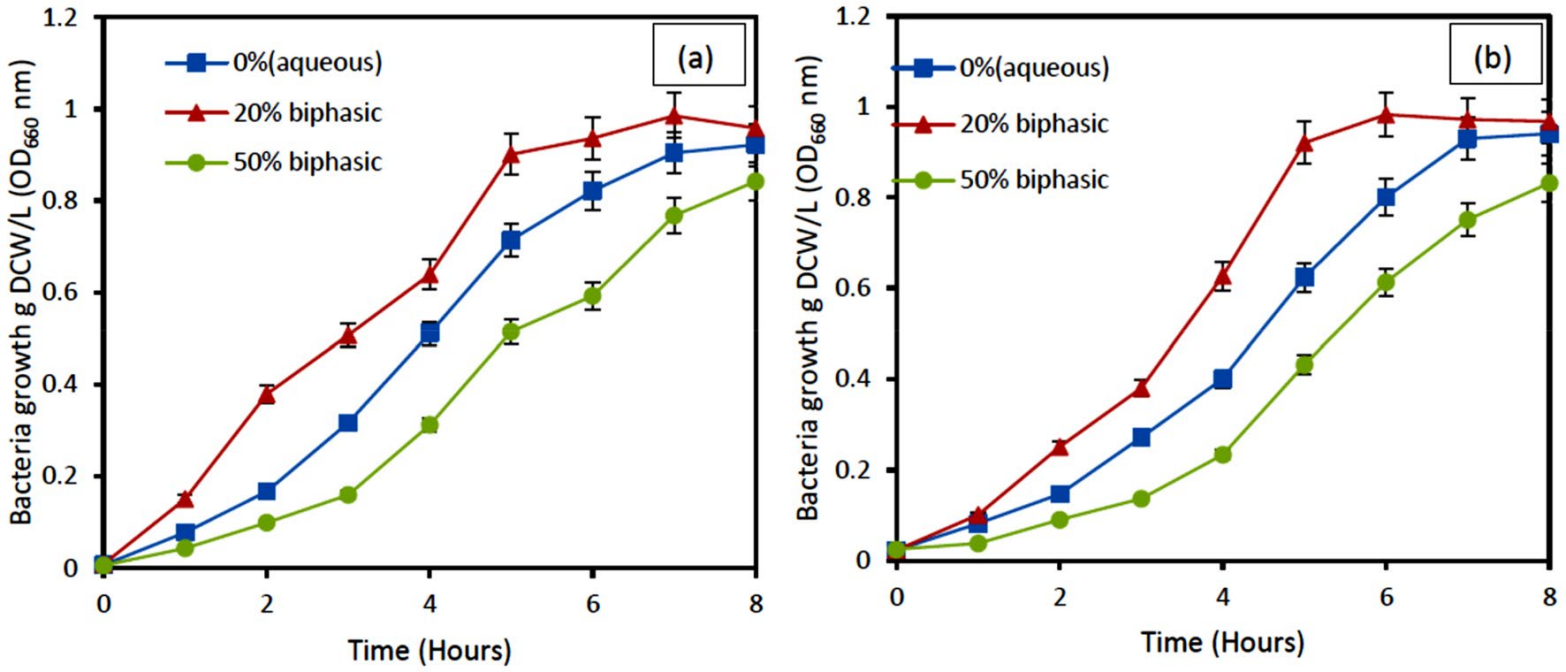
Effect of biphasic (oil–water ratio) on the growth of (**a**) *Pseudomonas aeruginosa* (**b**) *Pseudomonas Putida.* Experimental conditions: Initial DBT concentration of 500 ppm, Temperature 37 °C (*Pseudomonas aeruginosa*) 30 °C (*Pseudomonas putida*), Shaking speed 130 rpm.

Figure 7(a & b) described the effect of biphasic and aqueous medium on the growth of bacteria *Pseudomonas aeruginosa* and P*seudomonas putida*, respectively. It could be observed from the result in Fig. 7 that the optical densities of both *Pseudomonas aeruginosa* and P*seudomonas putida* in g DCW/L decreased with an increase in the percentage of oil to water at 50%. Results showed the best growth rate when a 20% oil phase was used compared to an aqueous medium without the oil phase and a 50% oil phase. The lower growth rate at 50% could be as a result of hydrophilicity of DBT, owing to the reduced concentration of DBT when the oil phase increased because the same initial concentration of DBT was used in all. It is assumed that the transfer of DBT from the oil phase to the aqueous phase is an important parameter especially when a biocatalyst that has lower capability to adhere at the interface is used^30^. Another reason this could be so, is that, there might be mass transfer limitation of DBT from the oil phase to the aqueous phase where the cells are present. It has however been discovered that the cells use DBT as its only sulfur source for it metabolic growth, since bacteria use the DBT in the model oil for its growth. Furthermore, it could mean that there was lower supply of oxygen as the oil phase increased. This result is consistent with Caro et al.^35^.

**Figure 8:**
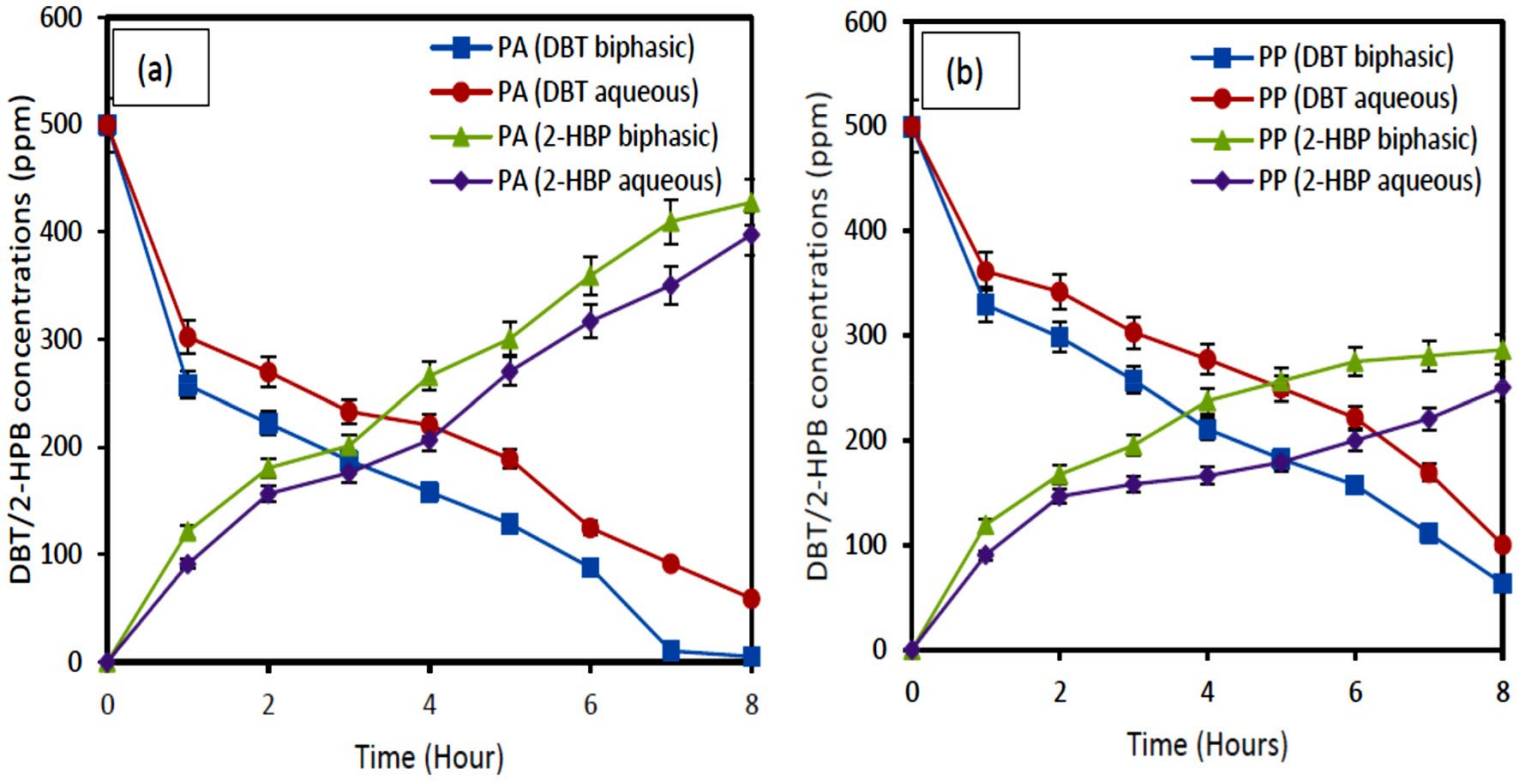
Effect of biphasic media on biodesulfurization of DBT and formation of 2-HBP by (**a**) *Pseudomonas aeruginosa* (**b**) *Pseudomonas putida.* Experimental conditions*:* Oil-to-water ratio *1:4.* Initial DBT concentration 500 ppm, Temperature 37 °C (Pseudomonas aeruginosa) 30 °C (Pseudomonas putida); shaking speed 130 rpm.

Figure 8(a & b) depict the effect of biphasic media on the biodesulfurization of DBT by *Pseudomonas* aeruginosa *and Pseudomonas putida*. The oil–water ratio is an essential factor in defining the reactor productivity and hence the reactor volume. The volume ratio of oil to water (O/W) affects the bioavailability of DBT when biodesulfurization occurs in the interface between the organic and the aqueous phases. Results showed better DBT degradation of 2-HBP production in the biphasic medium than in the aqueous medium. This could be as a result of better growth achieved in the result as discussed in Fig. 7. Enhanced desulfurization was achieved with resting cells of *Pseudomonas* aeruginosa *and Pseudomonas putida* in biphasic media compared to aqueous media. Hence, the amount of 2-HBP production was more than in the aqueous phase for both bacteria. This might be attributed to substrate availability and reduced product inhibition since 2-HBP which causes feedback inhibition is in the organic phase. The inhibition effect of 2-HBP was therefore avoided by channeling the DBT compound to the organic phase allowing the desulfurization process to continue unhindered in the aqueous phase. These results support the reports of a few researchers^29,36,37^.

### Results of model diesel compared with literature

Table 3 illustrates the results obtained in this study compared with the literature. Mingfang et al.^26^ utilized resting cells of Lyophilized R-8 to degrade DBT in model oil with an initial concentration of 1807 ppm. The result showed 55.23% BDS performance at a BDS rate of 8.75 Mm (g DCW/L)^−1^ h^−1^. Comparing this with the result obtained in this study, when the 500 ppm initial DBT was in a model oil, a BDS performance and BDS rate of 67.53% at 21.25 mM (g DCW/L)^−1^ h^−1^, respectively by resting cells of *Pseudomonas aeruginosa* and 50.02 ppm and 13.90 mM (g DCW/L)^−1^ h^−1^ by resting cells of *Pseudomonas putida* were obtained. The BDS performance results obtained in this study are better than what was obtained by Mingfang et al.^26^. This could be due to a higher initial DBT concentration used in their study*.* Alcon et al.^38^ desulfurized DBT in a model diesel using *Pseudomonas putida CECT 5259* for 10 h with an initial DBT concentration of 1.84 ppm. About 86% desulfurization efficiency was achieved by the bacteria. The result obtained in their study was higher than the result obtained in this study. This could be attributed to the higher initial DBT concentration of 120 ppm used in this study compared to the 1.84 ppm used by Alcon et al.^38^.

**Table 3:** Results of model diesel compared with literature.

Bacteria	Sulfur compound	Reaction time (h)	Initial DBT conc (ppm)	Final DBT conc (ppm)	% BDS	DZ rate (mM g (DCW/L)^-1^ h^-1^	Ref.
*Lyophilized cell of R-8*	DBT	24	1807	808.9	55.23	8.75	^26^
*PP CECT 5279*	DBT	10	1.84	0.26	86.00	0.12	^38^
*Pseudomonas Putida*	DBT	8	500	250.1	50.02	13.90	This study
*Pseudomonas aeruginosa*	DBT	8	500	162.4	67.53	21.25	This study

### Biodesulfurization performance of resting cells of *Pseudomonas aeruginosa* and *Pseudomonas putida* on real diesel samples

**Figure 9:**
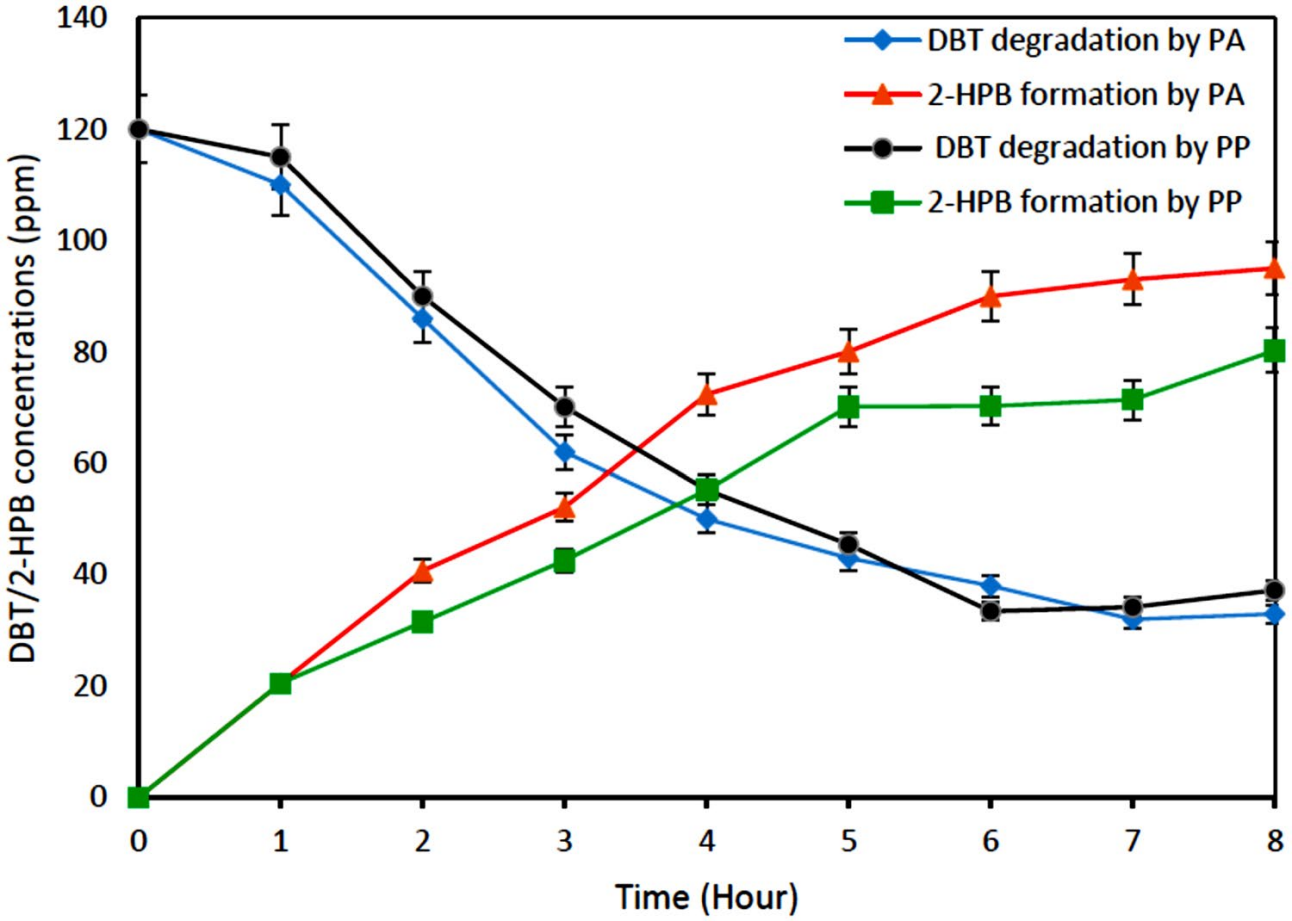
Biodesulfurization of diesel sample obtained after HDS (120 ppm) Experimental conditions: Cell centration 1.2 g DCW/L, Temperatures 30 °C and 37 °C for resting cells of *Pseudomonas putida* and *Pseudomonas aeruginosa*, respectively, Shaking speed 130 rpm.

Results of biodesulfurization of real diesel samples obtained from a typical South African refinery are depicted in Fig. 9. It illustrates the desulfurization of the diesel sample after HDS with an initial DBT concentration of 120 ppm. The results show that there was an appreciable increase in degradation of diesel sample after HDS from an initial DBT concentration of 120 ppm to 35.35 ppm for PA and from 120 ppm DBT to 38.99 ppm for PP. This accounted for about 70.54% and 67.50% degradation capacity for *Pseudomonas aeruginosa and Pseudomonas putida*, respectively. It could be observed as well that 36% of 2-HBP was produced when *Pseudomonas aeruginosa* was used as biocatalyst and 33% of 2-HBP was formed for biodesulfurization of diesel by *Pseudomonas putida*. This result is low when compared to what was obtained in the biodesulfurization of model diesel. This could be because a lot of compounds are present in the real diesel which might have negatively affected the selectivity of DBT for biodegradation^39^.

**Figure 10:**
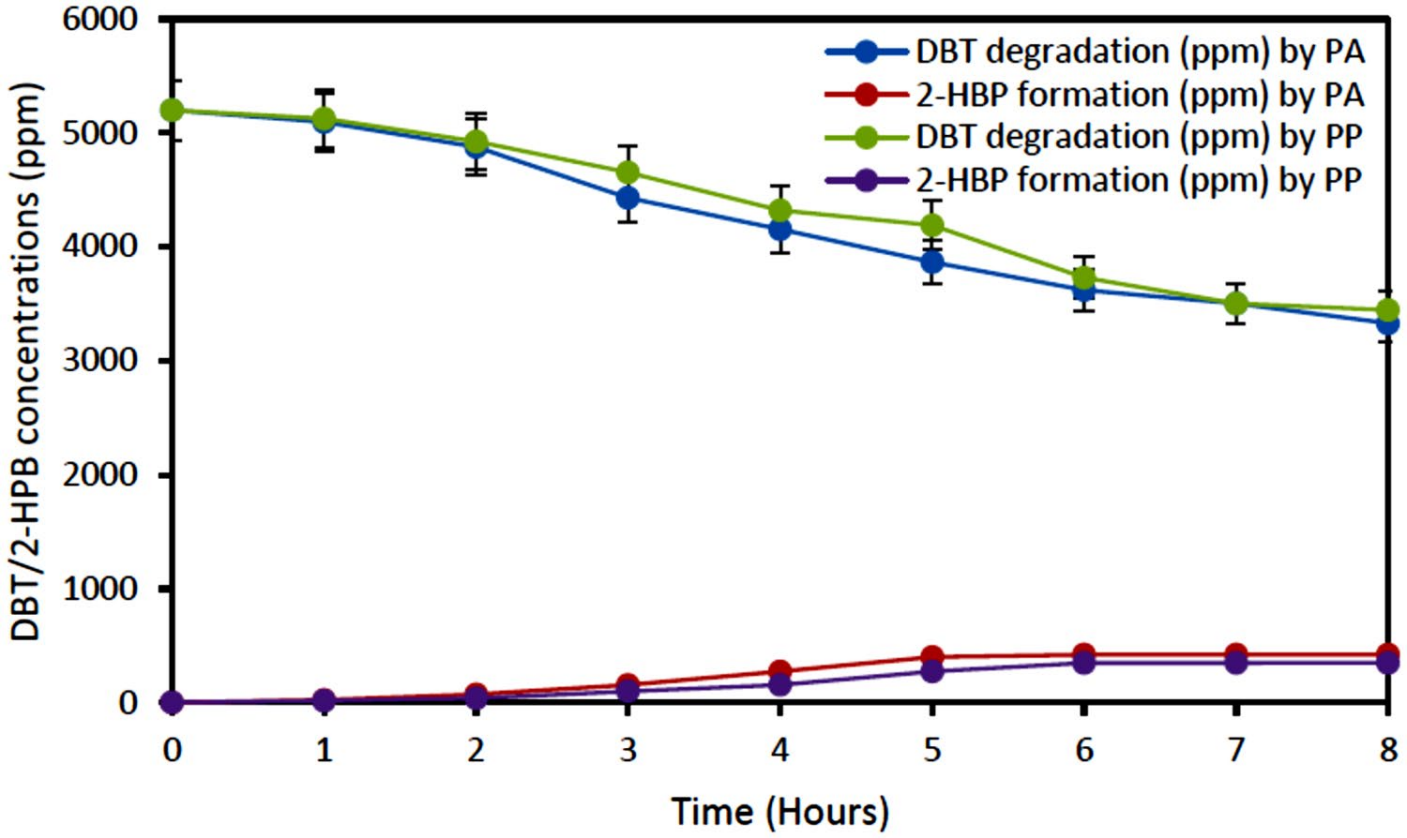
Biodesulfurization of diesel sample before HDS (5200 ppm). Experimental condition: Cell concentration 1.2 g DCW/L, Time 8 h, Temperatures 30 °C and 37 °C for resting cells of *Pseudomonas aeruginosa* and *Pseudomonas putida*, respectively, Shaking speed 130 rpm.

**Figure 11:**
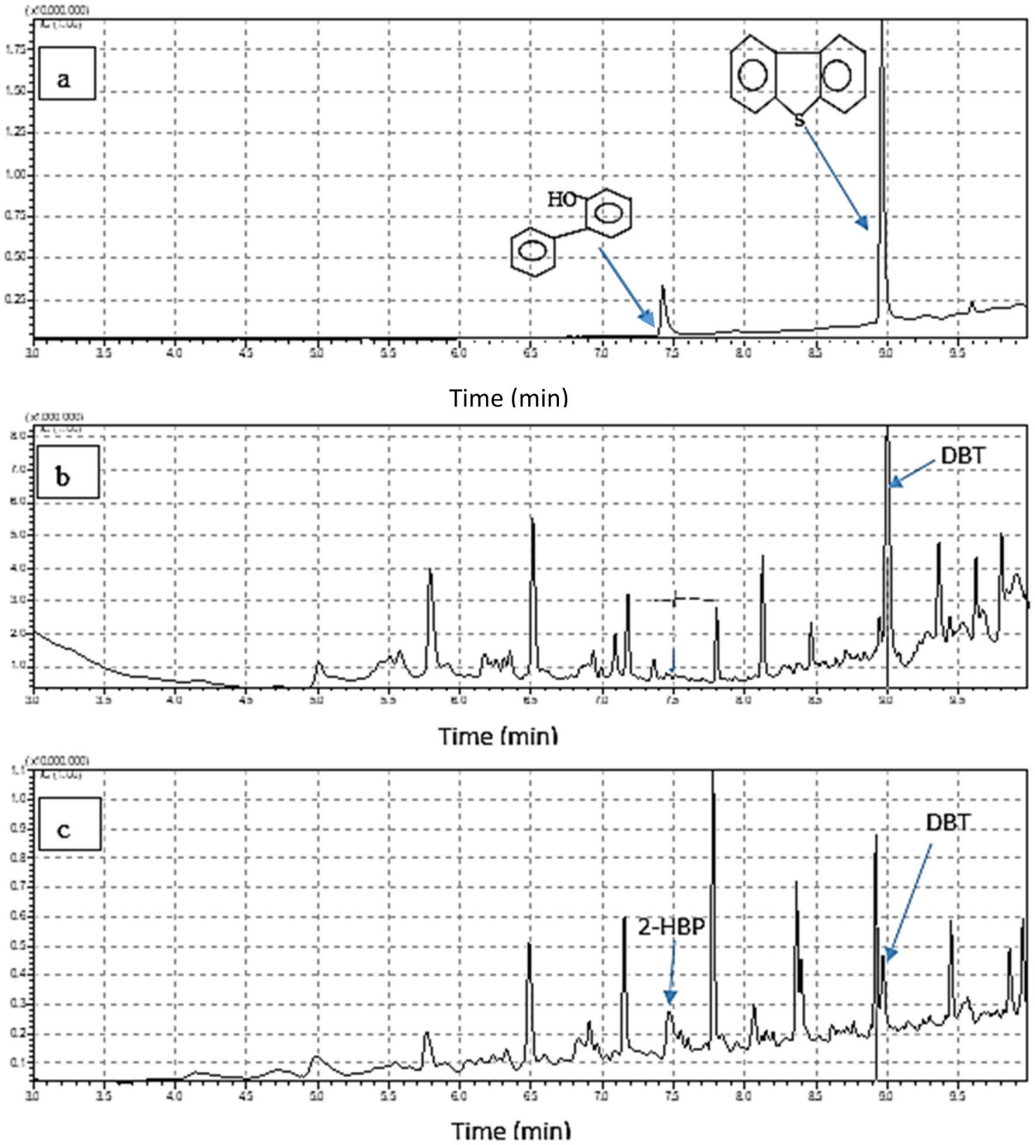
Analysis of diesel samples using GC/MS (**a**) standard DBT in hexane and standard 2-HBP in ethyl acetate, (**b**) real diesel sample obtained before hydrodesulfurization, (**c**) biodesulfurized real diesel sample.

Figure 10 depicts the desulfurization of DBT in diesel samples before HDS by *Pseudomonas aeruginosa and Pseudomonas putida,* with an initial DBT concentration of 5200 ppm. The results showed that about 36% and 33% desulfurization of diesel before HDS was achieved by *Pseudomonas aeruginosa and Pseudomonas putida*, respectively. After 8 h of biocatalyst activity at the resting stage, the diesel was shown to have reduced from its initial value (5200 ppm) to 3328 ppm for *Pseudomonas aeruginosa* and from 5200 to 3440 ppm for *Pseudomonas putida*. This low desulfurization performance could be attributed to the presence of other organosulfur compounds in the diesel (diesel sample obtained before HDS). In addition, high concentration of sulfur content could hinder the growth of bacteria (that is the concentration might be too toxic for the growth of the bacteria). This invariably affected the degradation efficiency of the DBT compound in the diesel. The formation of 2-HBP was also observed during the experiment as shown in Fig. 11b, although the production was significantly low (about 21.53% and 20.17% for *Pseudomonas aeruginosa and Pseudomonas putida*, respectively^39^.

**Figure 12:**
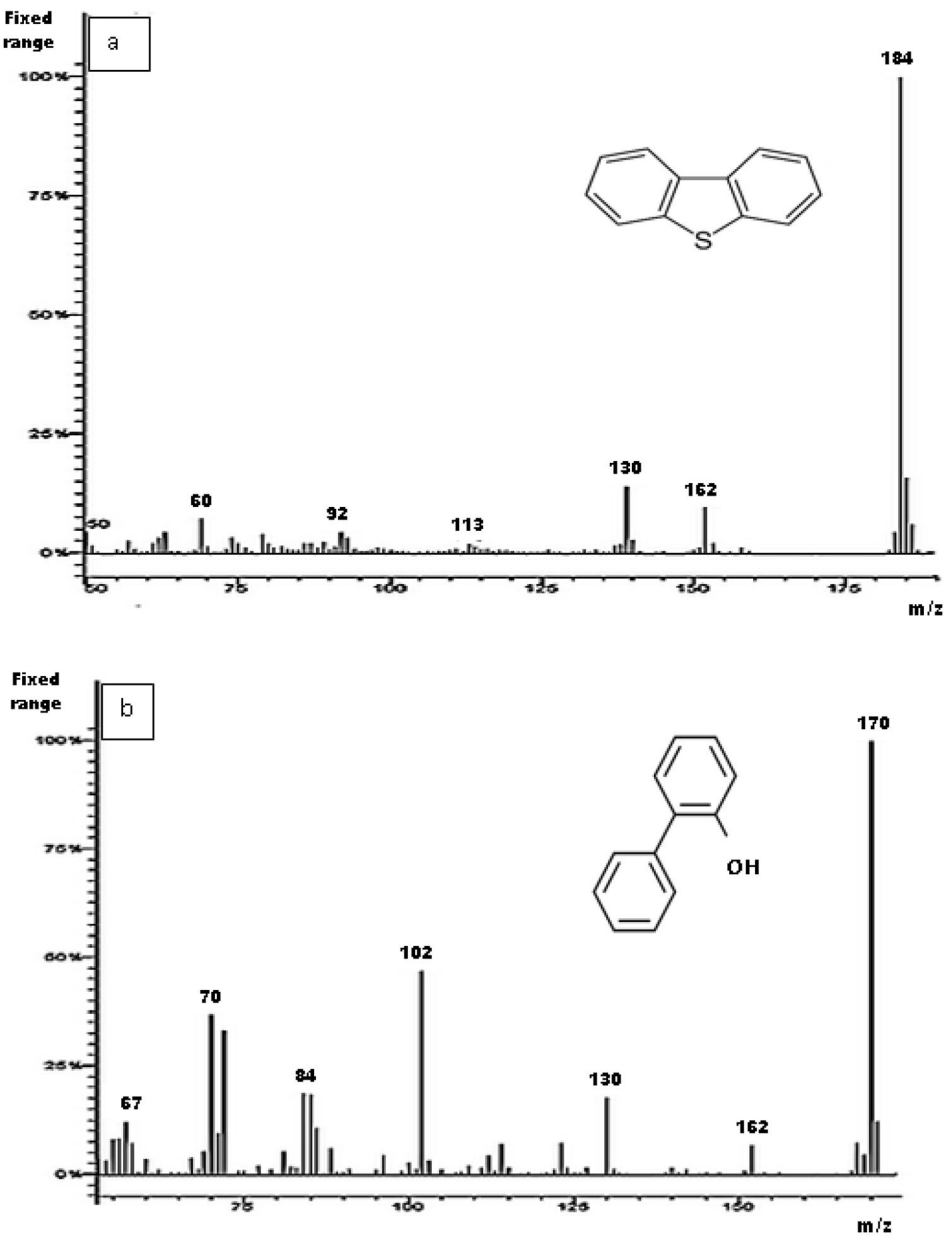
GC–MS mass spectra of (**a**) DBT (**b**) 2-HBP.

Figure 11(a to c) shows the GC/MS chromatograms of the standard DBT/2-HBP in hexane, the diesel obtained before biodesulfurization, and the biodesulfurized diesel, respectively. Figure 11a shows two peaks of two different retention times. This is an indication that the compound at a retention time of 9 min is DBT and at 7.5 min is 2-HBP. The chromatogram in Fig. 11c shows a decrease in the peak area of DBT when compared to that of the control sample before biodesulfurization in Fig. 11b. Also, there was an appearance of a peak at a retention time of 7.5 min (Fig. 11c) which indicates the formation of 2-HBP in the desulfurized diesel (real diesel). Figure 12a and b show MS spectra of DBT and 2-HBP at mass-to-charge ratio (m/z) 184 and 2-HBP at m/z 170, respectively^40^.

Table 4 shows the comparison of results obtained in this study with the literature. It could be observed that Chang et al.^17^, desulfurized middle distillate diesel using cells *Gordona CYKS1* for 10 h with an initial DBT concentration of 1500 ppm. The authors reported that the bacteria displayed a DBT degradation performance of 53%. The DBT biodegradation ability reported by the authors is higher than the DBT degradation ability of the *Pseudomonas aeruginosa* used in this study (about 30%). However, it should be noted that the initial concentration of 5200 ppm was used in this study as compared to 1500 pm used by Chang et al.^17^. The higher initial DBT concentration used in this study might have resulted in the lower DBT degradation performance of the bacteria as observed in this study. Higher DBT concentration could hamper the growth of the bacteria in the medium and subsequently result in lower consumption of DBT for growth. This speculation is evidently validated when a diesel sample obtained after HDS having initial DBT concentration of 120 ppm was used. With initial DBT concentration of 120 ppm, the biodegradation performance of the *Pseudomonas aeruginosa* was 70.54% and this result is higher than that of Chang and his co-authors^17^. Additionally, Chang et al.^17^ biodegraded the diesel sample for 10 h while 8 h was used in this study. In addition, their DBT in the middle distillate was degraded after 10 h, which is higher than 8 h, indicating that it could be possible that biodegradation performance of the *Pseudomonas aeruginosa* used in this study might be close to the value reported by Chang et al.^17^ if allowed for additional 2 h. Results reported in this article are comparable to the results of the investigation of Verma et al.^41^. The authors reported approximately DBT biodegradation performance of 88% with an isolate *E1 bacteria* when the initial DBT concentration was 368 ppm after 72 h biodegradation time. The shorter biodegradation time employed in this study (8 h) could have accounted for the lower biodegradation performance of *Pseudomonas aeruginosa* when compared to the results of Verma et al.^41^. Notwithstanding the disparities in the operating conditions of the reported literature when compared to the conditions employed in this study, results obtained in this study are comparable to the literature and could provide a platform for further research and development in this field. DBT metabolites such as DBTO, DBTO_2_ and HBPS were not detected in this study. Similarly, Silva et al.^34^ confirmed in their investigations that intermediate metabolites including DBTO, DBTO_2_, and HPBS were not discovered by GC–MS analysis, which might be explained by the existence of another pathway for the breakdown of DBT. Therefore, further investigations are required to identify all the metabolites in the 4S pathway.

**Table 4:** Results of desulfurization of real diesel compared with literature.

Biocatalyst	Sulfur compound	Reaction time (h)	Initial DBT conc (ppm)	Final DBT conc (ppm)	% BDS	DZ rate (mMg (DCW/L)^−1^ h^−1^	Ref.
*Gordona CYKS1*	Middle distillate	10	1500	610.0	59.3	94.00	^17^
*Isolate E1*	Crude oil	72	368	18.4	88.0	–	^41^
*Pseudomonas aeruginosa*	Hydro-treated diesel	8	120	15.0	70.5	28.67	This study
*Pseudomonas aeruginosa*	Diesel feed stock	8	5200	3444	30.0	12.96	This study

## Conclusions

The Pseudomonas strains were successfully grown and utilized for desulfurization of synthetic and real diesel in this study. *Pseudomonas aeruginosa* showed better BDS performance than *Pseudomonas putida* in all experiments. The results in this study showed that *Pseudomonas aeruginosa* and *Pseudomonas putida* can desulfurize DBT into less harmful compound, 2-HBP. However, further studies are required to determine their desulfurization efficiency for other sulfur organic compounds in real diesel.

The final product, 2-HBP, detected shows that the specific activity of DBT desulfurization is 4S –pathway. However, more investigations are still required in this field to detect all the various metabolites in the 4S pathway. In addition, the introduction of sufficient oxygen could improve the biodesulfurization performance of these bacteria cells.

In a nutshell, the study has shown that *Pseudomonas aeruginosa* and *Pseudomonas putida* could be better catalysts for the desulfurizing sulfur-containing compounds in South African diesel. The use of BDS has showcased in this study, in addition to HDS, could pave the way for the development of a hybrid process for the desulfurization of diesel. For future studies, a supply of oxygen into the bacteria medium may be required to enhance the growth of the bacteria, thereby enhancing the biodesulfurization efficiency. Furthermore, a thorough exploration into the understanding of the different microbial pathways that are involved in BDS may be required for the optimization and scale-up studies of the process.

## Data Availability

All data underlying the results are available as part of the article and no additional source data are required.
